# Spontaneous reduction of transvaginal small bowel evisceration after abdominal hysterectomy for cervical cancer: A case report

**DOI:** 10.1097/MD.0000000000029225

**Published:** 2022-05-06

**Authors:** Dong Hyung Lee, Eun Taeg Kim, Hyun Been Jo, Seo Yoon Hwang, Nam Kyung Lee, Dong Soo Suh, Ki Hyung Kim

**Affiliations:** a Department of Obstetrics and Gynecology, Pusan National University School of Medicine, Busan, Republic of Korea; b Biomedical Research Institute, Pusan National University Hospital, Busan, Republic of Korea; c Research Institute for Convergence of Biomedical Science and Technology, Pusan National University Yangsan Hospital, Busan, Republic of Korea; d Department of Radiology, Pusan National University School of Medicine, Busan, Republic of Korea.

**Keywords:** hysterectomy, spontaneous reduction, transvaginal small bowel evisceration

## Abstract

**Rationale:**

Transvaginal evisceration of the small bowel is an extremely rare condition after hysterectomy, which requires urgent surgical intervention to prevent serious bowel morbidity and mortality.

**Patient concerns:**

A 65-year-old woman presented with sudden-onset severe abdominal pain and a mass protruding through the vagina. The past surgical history was significant, with an abdominal hysterectomy for cervical cancer performed 11 weeks prior to presentation.

**Diagnosis:**

Pelvic examination revealed prolapsed small-bowel loops (18-20 cm in length). Pelvic computed tomography scan confirmed the presence of transvaginal evisceration of the small bowel.

**Interventions:**

Bowel reduction and urgent laparotomy were the selected treatment approaches for a detailed inspection and thorough washing of the intrα-abdominal cavity. A Foley catheter was inserted in the emergency room, with the subject in the lithotomy position. The prolapsed bowel loops spontaneously reduced without manual reduction, and the vault defect was repaired transvaginally.

**Outcomes:**

The patient experienced no postoperative complications and remained disease-free for 9months postoperatively.

**Lessons:**

Transvaginal evisceration of the small bowel should be considered a surgical emergency. A multidisciplinary approach to prompt case management involving clinicians in gynecology, general surgery, and emergency medicine is vital for preventing serious consequences. Hysterectomy is the most frequently performed gynecological surgical procedure, and evisceration occurs most often after hysterectomy. Therefore, patients should be informed about this rare but possible hysterectomy complication.

## 1 Introduction

Transvaginal evisceration of the small bowel is an exceedingly rare condition considered a potentially life-threatening surgical emergency. Possible risk factors for this condition include advanced age, previous pelvic surgery, obesity, constipation, cough, and radiotherapy.^[1,2]^ Prompt surgical treatment is necessary to prevent serious complications, such as bowel ischemia, strangulation, and abdominal sepsis. Delayed treatment increases morbidity and mortality. Physicians should be aware of this rare complication and immediate surgical management should be provided to salvage the prolapsed bowel. The viability of the eviscerated bowel should be assessed immediately and thoroughly when deciding the appropriate surgical route. Various approaches have been proposed for achieving this goal. Manual reduction can be performed when bowel viability is assured and repair is completed vaginally. A combined laparoscopic and vaginal approach allows surgeons to thoroughly inspect bowel length.^[3]^ In cases of suspected ischemic bowel injury or bowel strangulation, an abdominal approach is required to explore the abdominopelvic viscera, reduce the prolapsed bowel, and close the vaginal defect. Herein, we report the successful management of small- bowel evisceration after hysterectomy for cervical cancer through a transvaginal approach following spontaneous reduction of evisceration.

## 2 Case report

**Figure 1:**
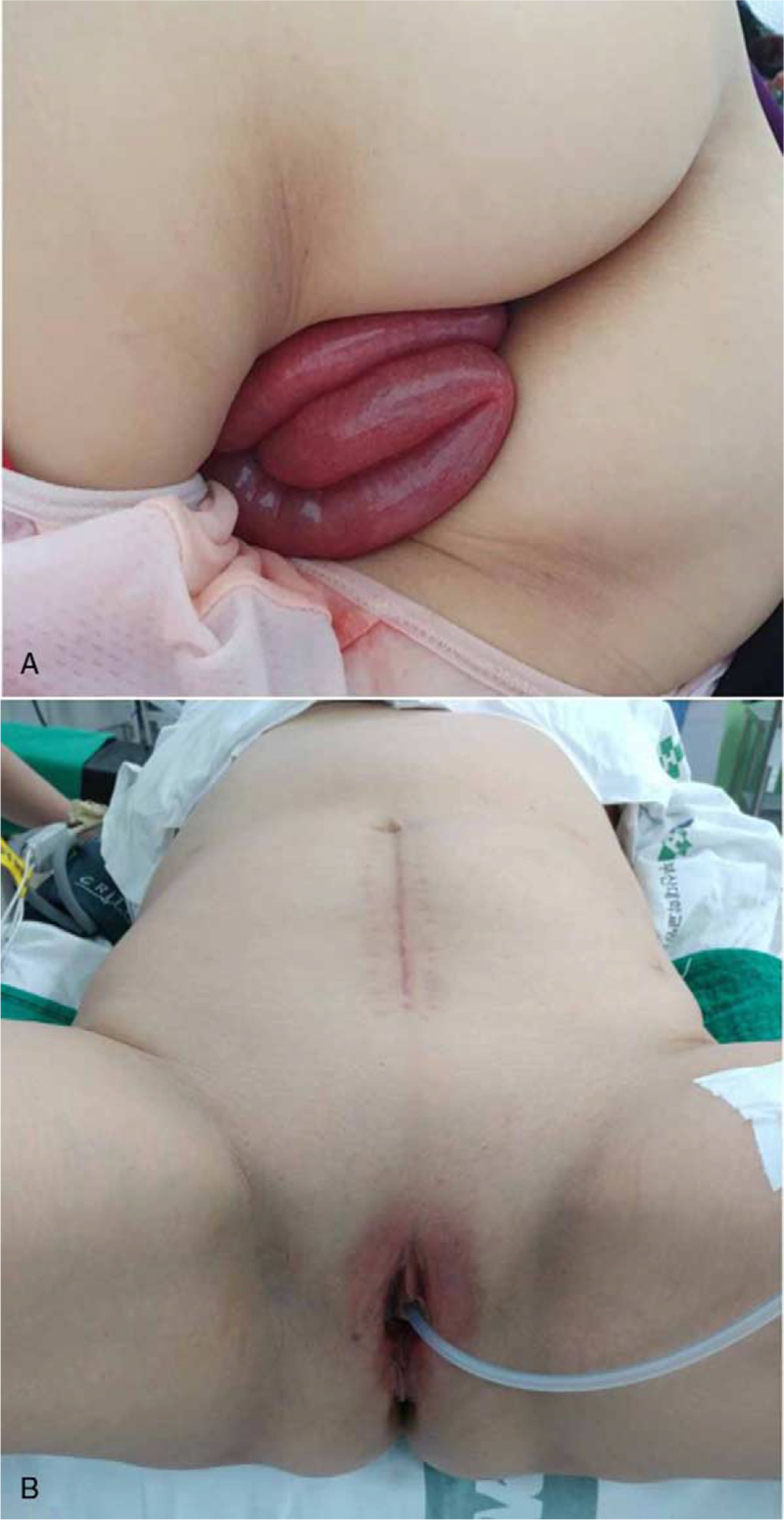
Transvaginal evisceration of the small bowel at presentation. (A) Eviscerated loops of the bowel show no signs of ischemia or necrosis. (B) Spontaneous reduction of bowel loop after insertion of a Foley catheter.

**Figure 2:**
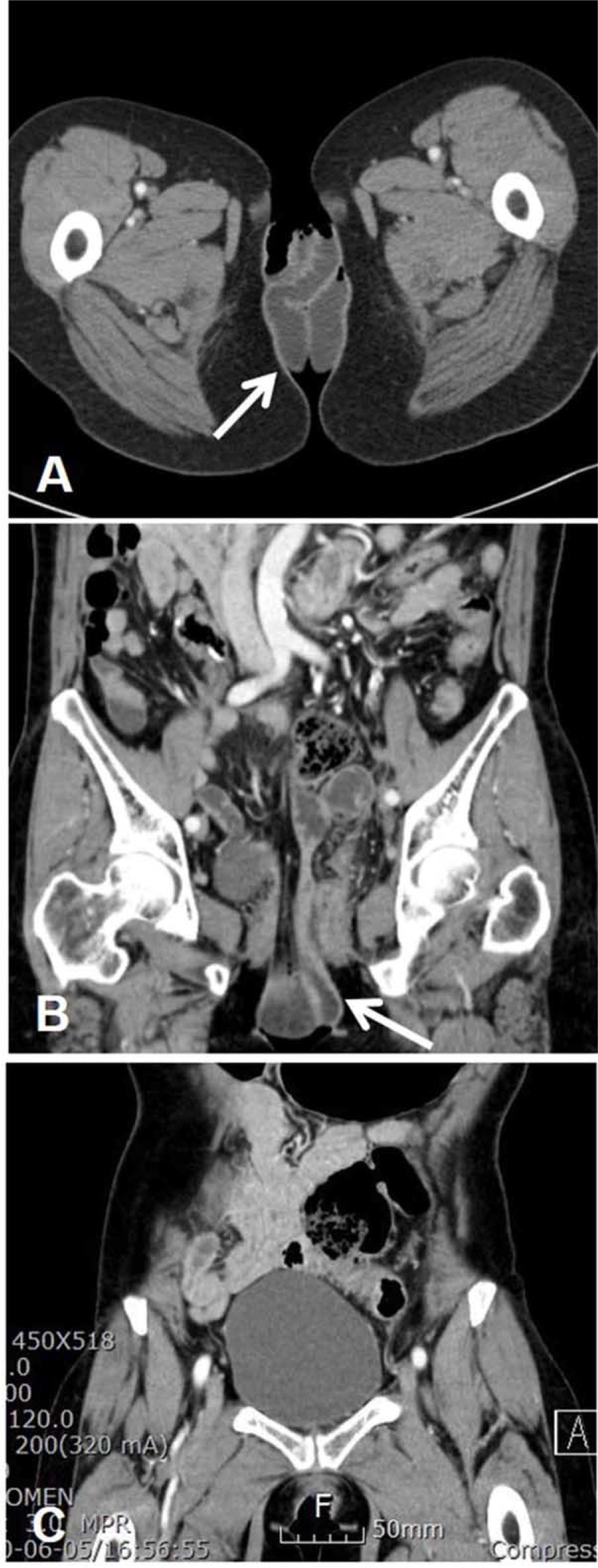
CT images of transvaginal evisceration of the small bowel. Axial (A) and coronal reformatted (B) contrast-enhanced CT images show herniation of small bowel loops (arrows) through the vaginal vault. (C) Distended bladder at presentation. CT = computed tomography.

A 65-year-old woman (gravida 3, para 2) presented with suddenonset severe abdominal pain lasting 1 hour, followed by a visible bowel loop protruding through the vagina. The patient reported that she experienced severe abdominal pain, nausea, lower back pain, sweating, and sudden appearance of a mass protruding through the vagina after sitting on her toilet for voiding. She visited the emergency room of the hospital immediately. Past surgical history was significant as she had undergone an abdominal modified-radical hysterectomy for micro-invasive cervical cancer (invasion depth 2 mm, horizontal spread 14 mm, no lymphovascular space invasion, IA1) 11 weeks before presentation at our institution and laparoscopic surgery for early colon cancer 10 years ago at another institution. At the time of hysterectomy, colpotomy was performed using scissors rather than using monopolar energy. Cuff closure was performed using 1-0 Vicryl sutures. The peritoneum around the vaginal cuff was sutured. Pelvic examination revealed prolapsed small-bowel loops (18–20 cm in length) (Fig. 1). The prolapsed bowel loops were pink in color and appeared viable. She had a history of constipation. Postoperatively, she often went to a bathhouse, but denied recent sexual intercourse after hysterectomy. In the emergency room, her vital signs remained stable with no fever. Her blood pressure was 140/80 mm Hg, heart rate was 68/min, respiratory rate was 18 breaths/min, and body temperature was 36.0°C. Pelvic computed tomography (CT) confirmed the presence of transvaginal evisceration of the small bowel (Fig. 2).

After the completion of the assessment, bowel reduction and urgent laparotomy were the selected treatment approaches for detailed inspection and thorough washing of the intra-abdominal cavity. A Foley catheter was inserted in the emergency room, with the subject in the lithotomy position. Unexpectedly, the prolapsed bowel loops spontaneously reduced without manual reduction. After transferring the patient to the operating room, reduction of the bowel loops was maintained. In the operating room, a gap of approximate 3 to 4 cm gap in the vaginal vault was observed. No devitalized tissue was observed along the vaginal vault margins. The gap was repaired transvaginally using 1-0 Vicryl interrupted sutures. The patient was discharged on postoperative day 2. Nineteen months postoperatively, the patient remained disease-free.

## 3 Discussion

Transvaginal small-bowel evisceration is a potentially lethal and urgent surgical condition. When small-bowel evisceration develops, urgent surgery is necessary to prevent subsequent fatal outcomes. This may lead to bowel injury, necrosis, sepsis, and death (mortality up to 8%).^[4]^ Fifteen percent to 20% of these patients require bowel resection. Small bowel evisceration usually occurs in postmenopausal women (70%) and in women with previous vaginal or uterine surgery, and is associated with decreased vascularity and atrophy of the vaginal wall. In this case, the patient was a postmenopausal woman with a recent history of hysterectomy for cervical cancer.

Risk factors for this event are advanced age and, pelvic surgery such as hysterectomy, obesity, enterocele, and radiotherapy, each of which is associated with weakening of the abdominal and vaginal walls.^[1,2]^ Precipitating factors include coitus, straining with defecation, and increased intrα-abdominal pressure during the Valsalva maneuver. Spontaneous vaginal cuff dehiscence or evisceration has been reported in up to 70% of the cases.^[5,6]^ Studies have shown that the incidence of vaginal cuff dehiscence following hysterectomy, a severe and potentially detrimental complication, varies with surgical approach. Recently, cuff dehiscence has increased significantly since the introduction of laparoscopic hysterectomy. The increased use of minimally invasive hysterectomies is a significant risk factor. Compared to other hysterectomy routes, laparoscopic and robotic hysterectomies are associated with a higher incidence of cuff dehiscence.^[7]^ The increased vaginal cuff dehiscence rate associated with laparoscopic hysterectomy was not due to electrosurgery, but rather to the vaginal closure technique.^[8]^ Furthermore, a systematic review of the literature indicated that transvaginal cuff closure after total laparoscopic hysterectomy is associated with a 3- and 9-fold reduction in the risk of vaginal cuff dehiscence compared with laparoscopic and robotic sutures, respectively.^[9]^ This study could serve as a basis for future investigations aimed at evaluating the impact of surgical techniques on the risk of vaginal cuff separation after minimally invasive hysterectomy. A recent systematic review and metaanalysis evaluating vaginal dehiscence and possible prevention strategies in the current literature after laparoscopic and robotic hysterectomy reported that the use of barbed sutures and adoption of a laparoscopic approach to close the vaginal cuff were effective.^[10]^ The incidence of transvaginal evisceration after hysterectomy ranges from 0.09% to 0.28%.^[11,12]^ Evisceration occurs in 35% to 67% of vaginal cuff dehiscence cases.^[3,11]^ The time interval between hysterectomy and evisceration varies considerably depending on the type of hysterectomy, and the median time to evisceration is 3, 6.5, and 34 months after laparoscopic, abdominal, and vaginal hysterectomies, respectively.^[1,13]^ In this case, the patient was an elderly woman with a history of abdominal hysterectomy and laparoscopic colon cancer surgery. The patient had no history of diabetes mellitus, long-term steroid use, or tobacco smoking, which are known risk factors for incomplete wound healing and vaginal cuff dehiscence. Her evisceration occurred 11 weeks postoperatively, which is much earlier than the reported median time of 6.5 months from abdominal hysterectomy to vaginal cuff dehiscence. An earlier occurrence than that in our case was reported in a 45-year-old woman with a history of total laparoscopic hysterectomy 8 weeks before its occurrence.^[13]^ In our case, the particular event triggering sudden bowel evisceration remains unclear. The patient had problems with recent bowel and hygiene habits, including longstanding constipation and frequent postoperative bathhouse douchings. These 2 factors may have weakened the vaginal vault and increased the intrα-abdominal pressure, leading to bowel evisceration. Postmenopausal vaginal atrophy may have contributed to this event.

Delays in the recognition and management of bowel evisceration can lead to serious morbidity and mortality. Different surgical alternatives, including abdominal, laparoscopic, vaginal, or combined laparoscopic-vaginal approaches, can be used to correct evisceration. The selection of the approach depends on the status of the eviscerated bowel and the condition of the vaginal cuff defect. A vaginal approach may be feasible when the bowel is easily reducible without evidence of complications. Although this approach has the advantage of being minimally invasive, its main drawback is that it restricts full bowel inspection. Emergency laparotomy for bowel exploration is required when transvaginal reduction of the bowel is unsuccessful, or when bowel viability is compromised. Ischemic and nonviable bowel tissues require resection. Therefore, the laparoscopic approach can be used as an alternative to the abdominal approach.^[12]^ A combined laparoscopic-vaginal approach has the additional benefit of allowing thorough inspection of the abdominal viscus.^[3]^

Spontaneous reduction of transvaginal small-bowel evisceration after hysterectomy, as observed in our case, has rarely been reported. We experienced spontaneous reduction of small bowel evisceration and subsequently performed transvaginal repair of the vault defect in an early evisceration case with no clinical evidence of peritonitis or bowel injury. However, we are unaware of the mechanisms by which spontaneous reduction occurs. The time from evisceration to reduction is likely the most important factor for a favorable outcome. In this case, unlike other cases, reduction occurred spontaneously approximately 1 hour after the evisceration event, and no bowel edema was observed. After hysterectomy, the axis of the vagina may change, making it either more vertical or shorter. Such a change may result in the vagina losing its valve-like structure.^[14]^ In this case, a distended bladder was observed on the computed tomography. This could be a chronic complication of radical hysterectomy for cervical cancer and might have interfered with spontaneous reduction of the prolapsed bowel. Bladder emptying following Foley catheter insertion may have facilitated spontaneous reduction of early stage evisceration in this patient. In addition, the use of the lithotomy-Trendelenburg position may have helped induce reduction.

In conclusion, transvaginal evisceration of the small bowel is exceedingly rare and should be considered as a surgical emergency. A multidisciplinary approach to prompt case management involving clinicians in gynecology, general surgery, and emergency medicine is vital to prevent the serious consequences of small bowel evisceration through the vagina. Hysterectomy is the most frequently performed gynecological surgical procedure, and evisceration occurs most often after hysterectomy. Therefore, patients should be informed about this rare but possible hysterectomy complication.

## Author contributions

**Conceptualization:** Dong Hyung Lee, Ki Hyung Kim.

**Resources:** Nam Kyung Lee, Hyun Been Jo.

**Supervision:** Eun Taeg Kim.

**Writing - original draft:** Dong Hyung Lee.

**Writing - review & editing:** Dong Soo Suh, Seo Yoon Hwang, Ki Hyung Kim.
